# Effects of aluminum on nucleoli in root tip cells and selected physiological and biochemical characters in *Allium cepa *var. *agrogarum *L

**DOI:** 10.1186/1471-2229-10-225

**Published:** 2010-10-21

**Authors:** Rong Qin, Yunqiu Jiao, Shanshan Zhang, Wusheng Jiang, Donghua Liu

**Affiliations:** 1 College of Life Sciences, Tianjin Normal University, Tianjin 300387, China; 2 Library of Tianjin Normal University, Tianjin 300387, China

## Abstract

**Background:**

Increased Al concentration causes reduction of mitotic activity, induction of nucleolar alteration, increase of the production of ROS and alteration of several antioxidant enzyme activities in plant cells. *Allium cepa *is an excellent plant and a useful biomarker for environmental monitoring. Limited information is available about the effects of Al on nucleoli, antioxidant enzyme system, contents of MDA and soluble protein in *A. cepa*. Therefore, we carried out the investigation in order to better understand the effects of Al on the growth, nucleoli in root tip cells and selected physiological and biochemical characters.

**Results:**

The results showed that the root growth exposed to 50 μM Al was inhibited significantly. 50 μM Al could induce some particles of argyrophilic proteins scattered in the nuclei and extruded from the nucleoli into the cytoplasm. The nucleolus did not disaggregate normally and still remained its characteristic structure during metaphase. Nucleolar reconstruction was inhibited. 50 μM Al induced high activities of SOD and POD in leaves and roots significantly (*P *< 0.05) when compared with control, whereas the level of CAT was low significantly (*P *< 0.05). At 50 μM Al the content of MDA in leaves was high significantly (*P *< 0.05) at 9^th ^day and in roots increased (*P *< 0.05) with prolonging the treatment time during 6-12 days. The soluble protein content in leaves treated with 50 μM Al was high significantly (*P *< 0.05) at 6^th ^day and increased with prolonging the treatment time.

**Conclusions:**

We suggest that variations in nucleoli and the alterations of antioxidant enzyme activities, MDA and soluble protein contents in *Allium cepa *can serve as useful biomarkers, which can provide valuable information for monitoring and forecasting effects of exposure to Al in real scenarios conditions. Among the antioxidant enzymes SOD and POD appear to play a key role in the antioxidant defense mechanism under Al toxicity condition. Data from MDA concentration show that Al indirectly produces superoxide radicals, resulting in increased lipid peroxidative products and oxidative stress.

## Background

Aluminum (Al) is the third most abundant element making up more than 8% of the earth's crust [1]. It is well known that Al is one of the most important factors limiting normal plant growth in acid soils. Al toxicity is manifested primarily by inhibition of root growth [2]. The root meristem is considered as one of the main sites of Al toxicity [3]. It is well demonstrated that Al is toxic to many plants at micromolar concentrations, affecting primarily the normal functioning of roots within minutes or hours of exposure of roots to Al [4].

Generation of reactive oxygen species (ROS) has been identified as an inevitable process of normal aerobic metabolism in plants and the four major types of ROS are singlet oxygen (^1^O_2_), superoxide (O_2_^-^), hydrogen peroxide (H_2_O_2_) and hydroxyl radical (OH^-^) [5]. ROS can damage biological molecules including DNA, RNA, protein and lipid by inducing peroxidation [6]. The results from some investigations have shown that Al stress can increase the production of ROS, and activate several antioxidant enzymes in plant cells [7], suggesting that Al stress might induce cell death in plants through ROS-activated programmed cell death [8]. There are protective enzymatic and non-enzymatic mechanisms to scavenge ROS and alleviate their deleterious effects in plants [9]. To resist oxidative stress, plants can induce a series of detoxification reactions catalyzed by antioxidant enzymes, including low-molecular mass antioxidants (ascorbic acid, glutathione and carotenoids) as well as CAT(EC 1.11.1.6), SOD (EC 1.15.1.1) and POD(EC 1.11.1.7)[10]. Lipid peroxidation occurs in plants as a consequence of high ROS level when excessive ROS can not be scavenged immediately and effectively, and finally resulting in the disruption of plant growth and development [11]. Malondialdehyde (MDA) is one of the ultimate products as a result of lipid peroxidation damage and its concentration is related to the degree of membrane lipid peroxidation [12]. Therefore antioxidant enzyme activities and MDA content often serve as important physiological indicators to research the resistant abilities of plants under stress conditions. Proteins play an important role in metabolism. There are several reports related to the change of soluble protein content under treatment with Al [13].

*Allium cepa *is well known and commonly used in many laboratories because *A. cepa *is an excellent plant and a useful biomarker for environmental monitoring, with many advantages such as low cost, a large number of roots, short test time, ease of storage and handling, large cells with excellent chromosome conditions, and ease of observing abnormal phenomena of chromosomes, nuclei, and nucleoli affected during mitosis [14]. Limited information is available about the effects of Al on nucleolus and antioxidant enzyme system and contents of MDA and soluble protein in *A. cepa*. For the present investigation, the effects of Al on root growth, nucleoli, activities of antioxidant enzymes, MDA and soluble protein contents in *A. cepa *were investigated to provide valuable information for monitoring and forecasting effects of exposure to Al in real scenarios conditions.

## Results

### Macroscopic effects of Al on root growth

**Figure 1:**
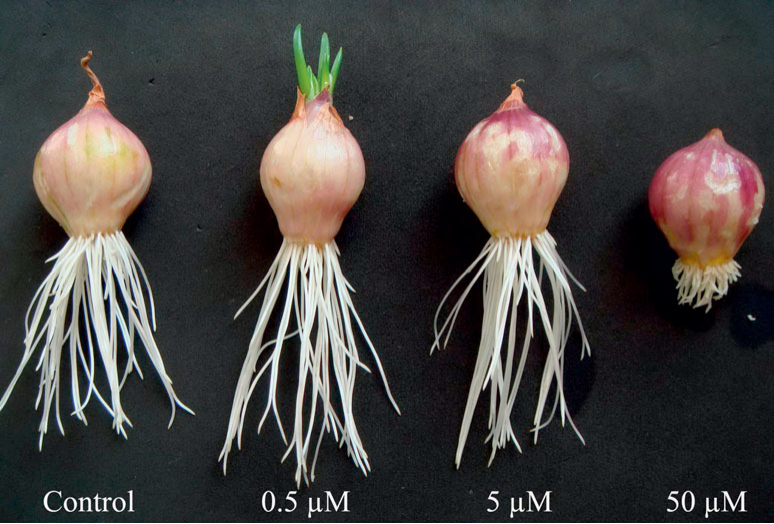
**Effects of different concentrations of Al on root growth of *Allium cepa *var. *agrogarum *L. (72 h)**.

**Figure 2:**
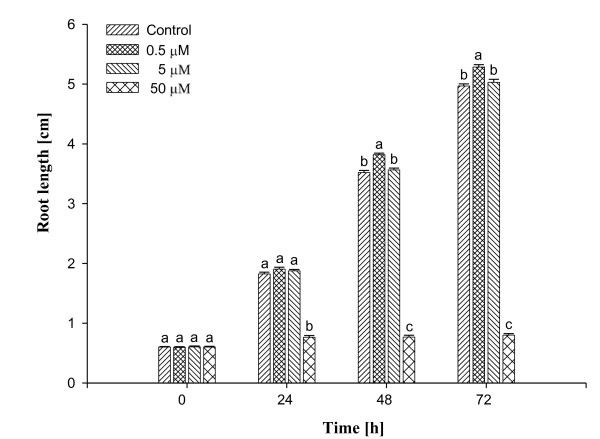
**Effects of different concentrations of Al on root length of *Allium cepa *var. *agrogarum *L. Values with different letters differ significantly from each other (n = 25, *P *< 0.05)**.

The effects of Al on root growth of *Allium cepa *var. *agrogarum *L. varied with the concentration and treatment time (Figures 1,2). At 5 μM Al there was no toxic effect on root growth during the whole course treatment. Versus control there was stimulative effect on root growth (*P *< 0.05) at 0.5 μM Al after 48 h treatment. In concentration 50 μM Al, obvious toxic effect appeared after 24 h treatment and Al inhibited root growth significantly (*P *< 0.05).

The effects of Al on the morphology of the roots also varied with the different concentrations of aluminum chloride in solution. At 0.5 μM to 5 μM Al, the morphology of the roots was more or less normal during the whole treatment (3 d). At 50 μM Al, the root tips were stunted and bent in various directions after 24 h treatment (Figure 1).

### Microscopic effects of Al on root tip cells

**Figure 3:**
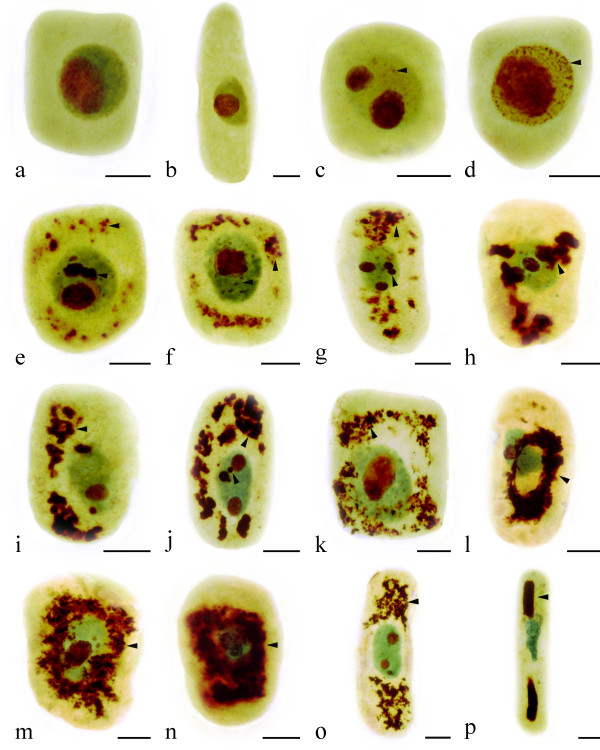
**Effects of different concentrations of Al on nucleoli in root tip cells of *Allium cepa *var. *agrogarum *L. (Arrowhead shows silver-stained materials)**. a-b. Control cells. c. Small amounts of silver-stained materials in nucleus (0.5 μM Al, 24 h). d. Large amounts of silver-stained materials in nucleus with increasing Al concentration and prolonging treatment time (5 μM Al, 48 h). e-f. Silver-stained materials extruded from the nucleus into the cytoplasm (50 μM Al, 24 h). g-k. Showing the leaching materials located near the nucleus and more and more materials accumulated in the cytoplasm with prolonging the duration of treatment (50 μM Al, 48 h). l-n. Showing the materials enclosed the nucleus, and accumulated in the cytoplasm and occupied nearly the whole cytoplasm (50 μM Al, 72 h). o-p. In long cells, the silver-stained materials gathered at the cell ends. (50 μM Al, 72 h) and large rod-like structures formed (50 μM Al, 72 h). Scale bar = 10 μm.

Normally, the nucleus of *Allium cepa *var. *agrogarum *contains one nucleolus (Figure 3a-b). The toxic effects of Al on nucleoli varied depending on the different concentrations and the treatment time. Some tiny particulates containing the argyrophilic proteins were observed in the nucleus of the root tips exposed to 0.5 μM Al for 24 h (Figure 3c). More particulates were accumulated in it with increasing Al concentration and prolonging treatment time, for example, at 5 μM Al, 48 h (Figure 3d). At high concentration of Al (50 μM), the effects were mainly on the nucleoli. The phenomenon was noted that some particulates containing the argyrophilic proteins were extruded from the nucleus into the cytoplasm in the group treated with 50 μM Al for 24 h (Figure 3e-f). The nucleolar materials accumulated in the cytoplasm gradually increased with prolonging the duration of treatment (Figure 3g-k). Figure 3g-k showed that the leaching materials were located near the nucleus. Finally, the material enclosed the nucleus and even occupied the whole cytoplasm (Figure 3l-n). In long cells, the nucleolar materials were extruded from the nucleus into the cytoplasm, gathered at the cell ends (Figure 3o) and large rod-like structures were formed (Figure 3p).

**Figure 4:**
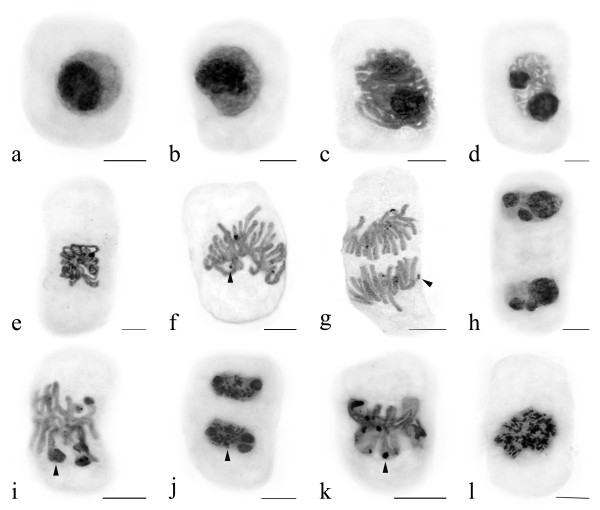
**Effects of Al on Nucleolar Organizing Regions (NORs) in root tip cells of *Allium cepa *var. *agrogarum *L. during mitosis (Arrowhead shows NORs)**. a-d. Showing decondensed chromatin fibers around the nucleoli. e. Showing decreased nucleoli in size. f. Showing NORs on chromosomes. g. Showing NORs migration with the chromosomes to the poles at anaphase. h. Showing nucleoli rebuilt at early telophase. i. Nucleoli still existed after the treatment with Al during metaphase (50 μM Al, 24 h). j. Showing some particles of the similar silver-stained materials localized in the nuclei (50 μM Al, 48 h). k. Showing nucleoli on chromosomes (50 μM Al, 48 h). l. Showing more similar silver-stained materials located on sticky chromosomes (50 μM Al, 72 h). Scale bar = 10 μm.

The nucleolar cycle of silver-impregnated *Allium cepa *var. *agrogarum *cells was investigated by means of light microscopy. Normally, nucleoli in interphase nuclei impregnated with silver show strong staining. With progressing prophase decondensed chromatin fibers were around the nucleoli (Figure 4a-d). During prometaphase-metaphase, the nucleoli appeared small in size (Figure 4e), disappeared in their characteristic structures and Nucleolar Organizing Regions (NORs) were localized on chromosomes (Figure 4f). At anaphase, NORs migrated with the chromosomes to the poles (Figure 4g). In early telophase the size of the newly forming nucleoli around the NORs increased (Figure 4h). Finally, mitosis was completed. After the treatment with Al, the abnormal phenomena of the nucleolar cycle during mitosis were examined in some cells. Firstly, the nucleoli did not disaggregate normally and still remained their characteristic structures during metaphase, which was referred to as persistent nucleoli (Figure 4i). Secondly, nucleolar reconstruction was inhibited, and there were still much small silver-stained particulates in the nuclei (Figure 4j). Thirdly, some particles of the silver-stained materials were still localized on chromosomes (Figure 4k). Fourthly, there were not NORs but lots of silver-stained particulates were localized on the sticky chromosomes (Figure 4l).

### Effects of Al on activities of antioxidant enzymes

**Figure 5:**
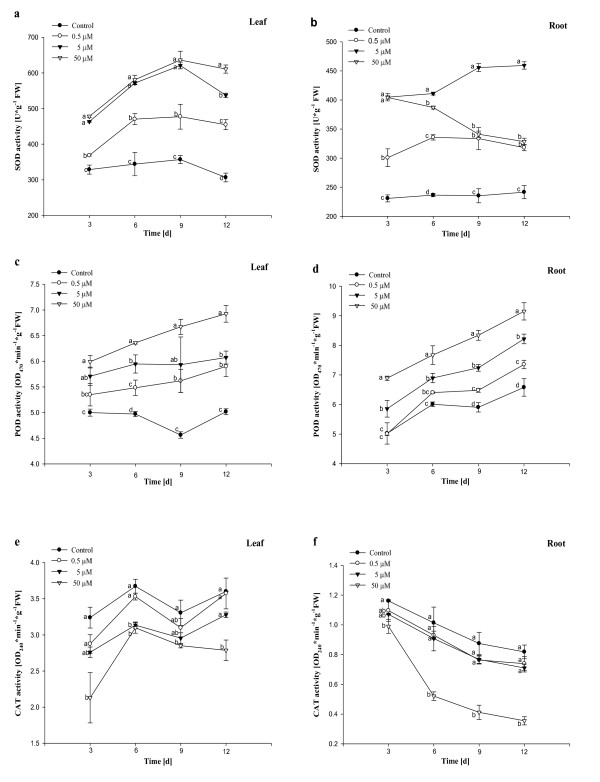
**Effects of different concentrations of Al on the activities of three antioxidant enzymes in *Allium cepa *var. *agrogarum *L. exposed to Al stress over 12 days**. a SOD in leaves, b SOD in roots, c POD in leaves, d POD in roots, e CAT in leaves, f CAT in roots. Vertical bars denote SE. Values with different letters differ significantly from each other (*P *< 0.05, *t*-test).

Effects of Al on SOD activities of *Allium cepa *var. *agrogarum *leaves and roots varied with the different concentrations of Al and the duration of treatment. The SOD activities in leaves exposed to 0.5 μM - 50 μM Al during the whole treatment were high significantly (*P *< 0.05) when compared with control (Figure 5a). And the levels of SOD in leaves treated with 5 μM - 50 μM Al were high significantly (*P *< 0.05) in comparison with the group exposed to 0.5 μM Al. The trend was observed that during 3 to 9 days, the SOD activities increased with prolonging treatment time, and then decreased. The level of SOD in the leaves exposed to 50 μM Al was 2 times that of control on the 12^th^ day. The activities in roots were lower than the ones in leaves (Figure 5a, b). Figure 5d showed the effects of different concentrations of Al on the SOD activities of *A. cepa *var. *agrogarum *roots. The levels of SOD in roots exposed to 0.5 μM - 50 μM Al were high significantly (*P *< 0.05) in comparison with control. The activity of SOD in roots treated with 5 μM Al was the highest and increased with prolonging the treatment time, whereas the activity at 50 μM Al decreased progressively.

Data from Figure 5c also showed the same trend observed in SOD that the levels of POD in leaves exposed to 0.5 μM - 50 μM Al were high significantly (*P *< 0.05) during the whole treatment when compared with control, except for the group exposed to 0.5 μM Al at 3^rd ^day. The activity of POD in leaves treated with 50 μM Al was the highest. The activities of POD in leaves exposed to all concentrations of Al increased with prolonging duration of treatment. The POD activity in roots treated with 50 μM Al was noted to be high significantly (*P *< 0.05) in comparison with control and the other treatment groups (Figure 5d). 0.5 μM Al had no obvious effect on the POD activity in roots during 3 - 6 days when compared with control (Figure 5d). The results indicated that the activity of POD in roots increased significantly with increasing Al concentration and prolonging treatment time, except the group exposed to 0.5 μM Al during 3 to 6 days.

Information on CAT activity was given in Figure 5e and f. The CAT activity was found to be inhibited significantly (*P *< 0.05) and to be the lowest in leaves at 50 μM Al during the whole treatment when compared with control and the group exposed to 0.5 μM Al (Figure 5e). There was no obvious difference in the CAT activities in leaves exposed to 0.5 μM Al and control during the whole treatment. The activity of CAT in leaves exposed to 5 μM Al was only inhibited significantly (*P *< 0.05) at 6^th ^day. There was an inhibitory effect (*P *< 0.05) on the CAT activity in roots treated with 50 μM Al in comparison with control and other treated groups during the whole treatment (Figure 5f). And the other groups had no obvious effects when compared with control.

### Effects of Al on MDA and soluble protein contents

**Figure 6:**
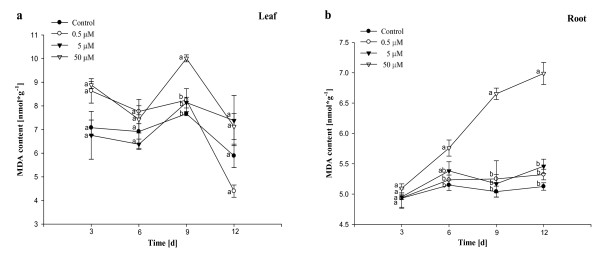
**Effects of different concentrations of Al on MDA content in *Allium cepa *var. *agrogarum *L. exposed to Al stress over 12 days**. a Leaves, b Roots. Vertical bars denote SE. Values with different letters differ significantly from each other (*P*< 0.05, *t*-test).

**Figure 7:**
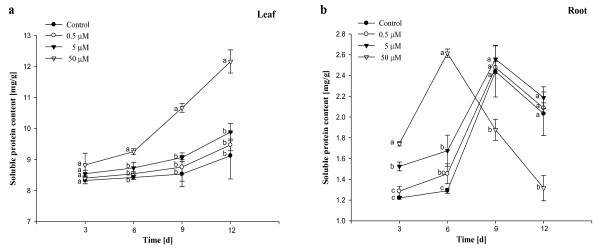
**Effects of different concentrations of Al on soluble protein content in *Allium cepa *var. *agrogarum *L. exposed to Al stress over 12 days**. a Leaves, b Roots. Vertical bars denote SE. Values with different letters differ significantly from each other (*P *< 0.05, *t*-test).

The effects of Al on MDA concentration were presented in Figure 6. The MDA contents in leaves exposed to 0.5 μM - 5 μM Al had no obvious difference when compared with control (Figure 6a). The content in leaves treated with 50 μM Al was high significantly (*P *< 0.05) at 9^th ^day. The MDA contents in roots exposed to 50 μM Al increased (*P *< 0.05) with prolonging the treatment time during 6-12 days when compared with control (Figure 6b). However, the MDA contents in roots treated with 0.5 μM - 5 μM Al were more or less the same as control (Figure 6b). As could be seen from Figure 7a, the soluble protein contents in leaves exposed to 0.5 μM -5 μM Al were more or less the same as control during the whole treatment time. The content in leaves treated with 50 μM Al was high significantly (*P *< 0.05) at 6^th ^day and increased with prolonging the treatment time. Data from Figure 7b showed that the content in roots exposed to 0.5 μM Al had no obvious difference when compared with control. The contents at 5 μM Al were high significantly (*P *< 0.05) at 3^rd ^day and 6^th ^day in comparison with control. The trend above was also observed at 50 μM Al. However, the content was low significantly (*P *< 0.05) from 9^th ^day and below control.

### Discussion

Root is the most sensitive and accessible part to Al toxicity, and root growth inhibition upon exposure to Al has been used extensively as one of the most distinct and earliest symptoms of Al toxicity [15]. Data from the present investigation demonstrated significant root growth inhibition in *Allium cepa *var. *agrogarum *L. seedlings exposed to 50 μM Al. This investigation showed that 0.5 μM Al had a stimulative effect on root growth which was in agreement with the early findings [16].

Nucleolus is well known as the site of transcription of ribosomal genes and further transcript process [17], which contains a set of acidic, nonhistone proteins that bind silver ions and are selectively visualized by silver method. NORs are defined as nucleolar components containing a set of argyrophilic proteins, which are selectively stained by the silver method [18]. Proteinic carboxyls firstly combine and deoxidize certain silver cations (Ag^–^), and then more silver cations continue to deposit at the focalization. So after silver-staining, the nucleoli can be selectively stained and the NORs can be easily identified as black dots [18].

Nucleolin is one of the main proteins in nucleolus and oxidative stress could induce the cleavage of it [19]. van der Aa et al. [20] indicated that the nuclear pore complex (NPC) was the most important channel for nuclear material. The phenomenon that the nucleolar material was extruded from the nucleus into the cytoplasm could be explained by the fact that the proteins were affected after Al treatment, causing the NPC to lose selectivity. Recent study indicated that Al was localized inside the nucleoli of root tip cells of Al sensitive maize [21].

Active NORs are associated with a subset of specific proteins and play an important role in forming nucleoli [22]. Normally, persistent nucleoli do not occur during normal mitosis. An increased number of persistent nucleoli cells increased nucleolar activity [23]. Sheldon et al. [24] found that embryonal carcinoma lines exhibited nucleolar persistence during mitotic metaphase and anaphase, indicating that rRNA synthesis continued in persistent nucleoli, which means increased biosynthetic activity and more protein production. We supposed that the phenomenon persistent nucleoli occurred in the present investigation might be an adaptive response to stress induced by Al.

Although Al itself is not a transition metal and cannot catalyze redox reactions, the Al-induced oxidative stress has been observed in many plant species [25]. It has even been suggested that Al-enhanced oxidative stress is a decisive event for inhibition of cell growth [26]. Al-induced oxidative stress and changes in cell wall properties have been suggested as the two major factors leading to Al toxicity [27]. The presence of oxygen in the cell environment can cause continuous oxidative damage to cell structure and function [28]. It is widely accepted that Al toxicity represents an oxidative stress in plants by inducing formation of ROS [8].

Superoxide dismutase is called the cell's first line of defense against ROS because superoxide radical is a precursor to several other highly reactive species so that control over the steady state of superoxide concentration by SOD constitutes an important protective mechanism [29]. SOD activity was affected by O_2_^- ^concentration and increased with the increasing of O_2_^- ^concentration [30]. Our results may be attributed to the increased production of superoxide, especially at concentrations at which root growth is strongly inhibited. The results here are consistent with the early findings [16,25]. The activity of SOD in roots treated with 50 μM Al decreased progressively with prolonging duration of treatment, which was similar to the early findings of Wang et al. [31]. This may be attributed to an inactivation of the enzyme by H_2_O_2 _produced in different compartments, where SOD catalyzes the disproportionation of superoxide radicals. Aravind and Prasad [32] indicated that excessive ROS could also attack SOD and decreased its activity.

Peroxidase activity increases with the increasing of H_2_O_2 _concentration [33] and protects cells against the destructive influence of H_2_O_2 _by catalysing its decomposition through oxidation of phenolic and endiolic co-substrates [8]. The enhanced activity of anionic POD could act to confer Al resistance by detoxifying ROS and restricting lipid peroxidation in membrane regions [34]. In the present investigation enhancement in POD activity was observed, which was in agreement with the observations by Hossain et al. [35] and Wang et al. [36]. Increase in POD activity was supposed that on the one hand Al directly caused excessive production of H_2_O_2 _in seedlings and on the other hand increased H_2_O_2 _was due to the result that SOD dismutated more O_2_^-^, subsequently excessive H_2_O_2 _induced the over-expression of POD gene. So increased POD activity, in turn, scavenged excessive H_2_O_2 _and the damage was not serious.

Catalase is the most universal oxidoreductase, which scavenges H_2_O_2_ to O_2_ and H_2_O. The major function of CAT is to metabolize the peroxide liberated in the peroxisome following the conversion of glycolate during photorespiration [37]. The CAT activities in leaves and roots treated with 50 μM Al in the present work declined whereas POD activities increased. It may be that POD plays a main role in clearing H_2_O_2_. Decline in CAT activity was supposed that it was due to inhibition of enzyme synthesis or a change in assembly of enzyme submits. Possibly CAT is a less efficient H_2_O_2 _scavenger than POD because of its low substrate affinity, and is more sensitive to high Al level than SOD and POD. Boscolo et al. [38] reported no change in CAT activity under Al toxicity in maize, while in some other plants a decline (soybean, rice) or enhancement (tobacco, wheat) of CAT activity has been found [39-41]. These results regarding CAT activity might be due to differences in the plant organs studied, the durations and concentrations of metals utilized, and the plant species.

In contrast, effects of Al on antioxidant enzymes are more serious in roots than in leaves, which can be explained by the fact that Al is taken up mostly through the root system, and accumulated high concentration in roots, only small amounts penetrate the leaves [42].

MDA formation is used as the general indicator of the extent of lipid peroxidation resulting from oxidative stress. Our results indicated that the extent of lipid peroxidation was not serious in leaves under 0.5 μM - 50 μM Al stress, suggesting that ROS was eliminated effectively as to the increase of antioxidant enzymes (SOD and POD) activities in leaves. MDA concentrations in roots exposed to 50 μM Al during 6-12 days increased versus control, indicating that Al indirectly produced ROS and there was a serious imbalance between the production of ROS and antioxidative defense, resulting in increased lipid peroxidative products and oxidative stress in roots.

In the present study, it was found that under 50 μM Al stress, soluble protein contents in leaves increased significantly (*P *< 0.05) during 6 to 12 day treatment. The result supports the findings by Özdemir et al. [43] and Zhou et al. [44]. The high soluble protein content induced by Al can be explained by the following two aspects. On the one hand Al induces the expression of several genes and increases the synthesis of several original proteins [45]. On the other hand Al-resistance proteins are inducible by high concentration of Al exposure [46]. In roots exposed to 50 μM Al at 3^rd ^day and 6^th ^day, the soluble protein contents were high significantly (*P *< 0.05). Then it showed a decreased. We considered that during earlier period of treatment, Al did not injure the roots heavily and induced protein synthesis and accumulation in cells. Al, with the stress strengthening, caused the original protein degeneration and decomposition [47] and restrained the new protein synthesis [48], which made soluble protein content decrease significantly.

### Conclusion

In view of the present findings, we suggest that (1) variations in nucleoli and alterations of antioxidant enzymes and MDA and soluble protein contents in *Allium cepa *can serve as useful biomarkers in ecotoxicological tests with Al; (2) These biomarkers can provide valuable information for monitoring and forecasting early effects of exposure to Al in real scenarios conditions; (3) Al toxicity is associated with induction of oxidative stress in leaves and roots of *A. cepa*. Among the antioxidant enzymes SOD and POD appear to play a key role in the antioxidant defense mechanism and (4) MDA concentration shows that Al indirectly produces superoxide radicals, resulting in increased lipid peroxidative products and oxidative stress in roots.

## Methods

### Culture condition and aluminum treatment

Healthy and equal-sized onion cloves were chosen from *Allium cepa *var*. agrogarum* L. The bulbs had started neither shooting of green leaves nor any growth of roots. Before starting the experiment, the dry scales of the bulbs were removed. The bulbs were germinated and grown in plastic containers at 27°C for 3 days by dipping the base in water. Then the seedlings were grown in containers with 2 L Hoagland's nutrient solution (pH 4.5) adding 0.5 μM, 5 μM and 50 μM Al for 12 days respectively in a greenhouse where relative humidity (60%) and supplementary lighting (14 h photoperiod) were controlled. The Hoagland's solution consisted of 5 mM Ca (NO_3_)_2_, 5 mM KNO_3_, 1 mM KH_2_PO_4_, 50 μM H_3_BO_3_, 1 mM MgSO_4_, 4.5 μM MnCl_2_, 3.8 μM ZnSO_4_, 0.3 μM CuSO_4, _0.1 μM (NH_4_)_6_Mo_7_O_24 _and 10 μM FeEDTA [49]. Hoagland's nutrient solution was used for control. The solutions were aerated by pumps, which connected the containers with pump lines. In each treatment group, twenty-four treated seedlings were examined and recorded every 24 h for the morphological observation (72 h) and for examination of antioxidant enzyme activities and MDA and soluble protein contents at the end of each time interval (3 d). All treatments were done in six replicates. The Al was provided as aluminum chloride.

### Cytological study

The bulbs were germinated in distilled water at 25°C, producing roots reaching about 0.6 cm length. After that, they were treated in Patri dishes with different concentrations of Al solutions (0.5 μM, 5 μM and 50 μM) for 24 h, 48 h and 72 h. Distilled water was used for control experiment. The test liquids were changed regularly every 24 h. Ten root tips in each treatment group were cut and fixed in 3 parts 95% ethanol:2 parts acetic acid for 2 h and hydrolyzed in 5 parts 1 M hydrochloric acid:3 parts 95% ethanol:2 parts 99.8% acetic acid for 4-5 min at 60°C. For the observation of changes in nucleolus, ten root tips were cut and squashed in 45% acetic acid, dried, and after 2 days stained with silver nitrate [50].

### Examination of antioxidant enzyme activities

The fresh roots or leaves from each treatment were homogenized in a pestle and mortar with 0.05 M sodium phosphate buffer (pH 7.8) at the end of each time interval (3 d) of the Al treatment. The homogenate was centrifuged at 10,000 × g for 20 min and the supernatant was used for analyzing SOD, POD and CAT. The above steps were carried out at 4°C [51].

### SOD assay

The SOD activity was estimated according to the modified method of Zhang et al. [52]. The reaction mixture was made of 54 mL methionine, 2 mL nitroblue tetrazolium chloride (NBT), 2 mL EDTA-Na_2_, 2 mL riboflavin. Appropriate quantity of enzyme extract was added to the reaction mixture. The reaction started by placing tubes below two 15 W fluorescent lamps for 15 min. Reaction stopped by keeping the tubes in dark for 10 min. Absorbance was recorded at 560 nm. One unit of SOD enzyme activity was defined as the quantity of SOD enzyme required to produce a 50% inhibition of reduction of NBT under the experimental conditions and the specific enzyme activity was expressed as units per g fresh weight.

### POD assay

The activity of POD was determined as described by Zhang et al. [52]. The reaction mixture in a total volume of 50 mL 0.1 M sodium phosphate buffer (pH 6.0) containing 19 μL H_2_O_2 _(30%), 28 μL guaiacol was prepared immediately before use. Then 1 mL enzyme extract was added to 3 mL reaction mixture. Increase in absorbance was measured at 470 nm at 0.5 min intervals up to 2 min using a UV-Vis spectrophotometer (UV-2550, Shimadzu, Kyoto, Japan). Enzyme specific activity is defined as units (one peroxidase activity unit defined as absorbance at 470 nm changes per minute) per g of fresh weight.

### CAT assay

CAT activity was assayed according to the method of Zhang et al. [52]. CAT activity was determined by a UV-Vis spectrophotometer (UV-2550, Shimadzu, Kyoto, Japan) in 2.8 mL reaction mixture containing 1.5 mL 0.05 M sodium phosphate buffer (pH 7.8), 1 mL deionized water and 0.3 mL 0.1 M H_2_O_2 _prepared immediately before use, then 0.2 mL enzyme extract was added. The CAT activity was measured by monitoring the decrease in absorbance at 240 nm at 0.5 min intervals up to 2 min as a consequence of H_2_O_2 _consumption. Activity was expressed as units (one catalase activity unit defined as absorbance at 240 nm changes per minute) per g of fresh weight.

### Examination of MDA content

Level of lipid peroxidation was expressed as the content of malondialdehyde (MDA) according to Zhang et al. [52]. The fresh samples from each treatment were homogenized in 5 mL of 10% trichloroacetic acid (TCA) with a pestle and mortar at the end of each time interval (3 d). Homogenates were centrifuged at 4000 × g for 20 min. To each 2 mL aliquot of the supernatant, 2 mL of 0.6% 2-thiobarbituric acid (TBA) in 10% TCA was added. The mixtures were heated in boiled water for 15 min and then quickly cooled in an ice bath. After centrifugation at 4000 × g for 10 min, the absorbance of the supernatant was recorded at 532 nm and 450 nm. Lipid peroxidation was expressed as the MDA content in nmol per g of fresh weight.

### Measurement of soluble protein content

Measuring soluble protein content in this investigation was carried out according to Bradford's method [53] using BSA as a standard. The fresh roots and leaves from each treatment (6 seedlings) were washed in distilled water, dried and put in a mortar with 5 mL 0.05 M PBS (pH 7.8) at the end of each time interval (3 d) of the Al treatment. The homogenate was centrifuged at 10,000 × g for 20 min and the supernatant was used for analyzing soluble protein content. The soluble protein content was expressed as mg per g fresh weight.

### Statistical analysis

Each treatment was replicated 6 times for statistical validity. Analysis of variance of the data was done with SigmaPlot 8.0 software. For statistical analysis, one-way analysis of variance (ANOVA) and *t*-test were used to determine the significance at *P *< 0.05.
